# Regional cerebellar atrophy related to disability and cognitive progression in multiple sclerosis

**DOI:** 10.1016/j.nicl.2025.103792

**Published:** 2025-04-23

**Authors:** Myrte Strik, Iris Dekker, Aurélie Ruet, Hanneke E. Hulst, Mike P. Wattjes, Frederik Barkhof, Bernard M.J. Uitdehaag, Joep Killestein, Menno M. Schoonheim

**Affiliations:** a Anatomy and Neurosciences, MS Center Amsterdam, Vrije Universiteit Amsterdam, Amsterdam Neuroscience, Amsterdam UMC Location VUmc, Amsterdam, the Netherlands; b Spinoza Centre for Neuroimaging, Amsterdam, the Netherlands; c Computational Cognitive Neuroscience and Neuroimaging, Netherlands Institute for Neuroscience, Royal Netherlands Academy for Arts and Sciences (KNAW), Amsterdam, the Netherlands; d Departments of Radiology and Nuclear Medicine, MS Center Amsterdam, Vrije Universiteit Amsterdam, Amsterdam Neuroscience, Amsterdam UMC Location VUmc, the Netherlands; e Departments of Neurology, MS Center Amsterdam, Vrije Universiteit Amsterdam, Amsterdam Neuroscience, Amsterdam UMC Location VUmc, the Netherlands; f Service de Neurologie, Pathologies inflammatoires du système nerveux central, CHU de Bordeaux, Bordeaux, France; g U1215 INSERM, Neurocentre Magendie, Université de Bordeaux, Bordeaux, France; h Institute of Psychology, Health, Medical and Neuropsychology Unit, Leiden University, Leiden, the Netherlands; i Department of Neuroradiology, Charité - Universitätsmedizin Berlin, Corporate Member of Freie Universität Berlin, Humboldt-Universität zu Berlin, Berlin, Germany; j Queen Square Institute of Neurology and Centre for Medical Image Computing, University College London, UK

**Keywords:** Cerebellum, Structural MRI, Multiple sclerosis, Disability, Cognition, Atrophy, Longitudinal

## Abstract

**Objective:**

The implications of cerebellar pathology on clinical disease progression in multiple sclerosis (MS) remain unclear. This study investigated regional cerebellar atrophy related to physical disability and cognitive impairment progression.

**Methods:**

We included 331 MS patients and 95 controls (Amsterdam MS Cohort, 229 patients and 58 controls re-evaluated after 5 years). Assessments included baseline MRI, and disability and cognition at baseline and follow up. Cerebellar (sub)cortex was parcellated, volumetric data were determined and related to baseline disability and cognition. Longitudinal progression was explored only for regions with significant baseline correlations.

**Results:**

At baseline, patients had mild disability (median EDSS 3.0) and 46% showing mild-to-severe cognitive impairment. At follow-up, 34.5% showed EDSS progression and 26.6% cognitive decline. All global and most regional volumes showed atrophy. Cross-sectionally, atrophy of several regions encompassing both anterior and posterior lobes correlated with both disability and cognition, while some correlated with EDSS only. Additionally, cerebellar nuclei only correlated with cognition. Cerebellar volumes were mainly related to information processing speed, working and verbal memory. Longitudinally, atrophy in the posterior lobe, lobule VI and VIIIb, and vermis VI, correlated with cognitive decline, while no variables correlated with disability progression.

**Conclusion:**

Regional cerebellar atrophy in both anterior and posterior lobes correlated with disability and cognitive impairment. Posterior regional atrophy was correlated with longitudinal cognitive decline, but none correlated with disability progression. Further research is required to elucidate these relationships.

## Highlights

• Regional cerebellar atrophy linked to clinical impairment in multiple sclerosis.
• Distinct and overlapping atrophy patterns related to disability and cognition.
• Posterior regional atrophy was correlated with longitudinal cognitive decline.
• Regional atrophy did not correlate with disability progression.

## 1 Introduction

Physical disabilities and cognitive impairments in multiple sclerosis (MS) have often been linked to damage in the cerebrum and spinal cord. However, the cerebellum is also frequently affected, including white matter damage as well as grey matter pathology affecting nearly the entire cortex (Calabrese et al., 2010, Kutzelnigg et al., 2007). The cerebellum is integral to major brain networks and plays therefore an important role in a range of functions ranging from motor control to cognitive processes (Guell et al., 2020, Stoodley et al., 2012, Weier et al., 2015). However, the cerebellum has been excluded frequently due to technical imaging challenges. Given the frequent MS damage in the cerebellum and its crucial clinical importance (Weier et al., 2015), further cerebellar research is needed.

While global cerebellar atrophy has been studied before, in-depth regional investigations of cerebellar atrophy are limited. Methodological difficulties specific to regional cerebellar segmentation have long been an issue as the cerebellar cortex is thinner and more tightly folded compared to the cerebrum. Previous research that focused on global measures indicated that atrophy of the anterior lobe (lobules I-V) is mainly related to disability and posterior lobe (lobules VI-IX) atrophy to cognitive performance (D’Ambrosio et al., 2016, Stoodley et al., 2012, Stoodley and Schmahmann, 2010). Recent methodological advances now allow for more reliable and sensitive parcellation of the cerebellum, enabling the analysis of regional atrophy patterns by subdividing the cerebellum into distinct lobules and nuclei (Diedrichsen, 2006, Diedrichsen et al., 2009, King et al., 2019). There is evidence that regional cerebellar atrophy is associated with physical disability in MS, (D’Ambrosio et al., 2016, Eijlers et al., 2017, Eijlers et al., 2018, Guell et al., 2020, Henry et al., 2008) and cognitive impairments such as information processing speed (Bonacchi et al., 2022, Cocozza et al., 2017, Moroso et al., 2017). However, the broad range of cognitive domains and the role of cerebellar nuclei remain underinvestigated. In addition, longitudinal studies assessing the predictive value of regional cerebellar atrophy on disability progression and cognitive decline are lacking.

Therefore, the aim of this study was to examine the cross-sectional relation between regional cerebellar atrophy, physical disability and cognitive impairment, to study cognitive domains and to explore the predictive value of regional cerebellar atrophy for longitudinal changes in disability and cognition in individuals with MS.

## 2 Methods

### 2.1 Participants

We included 331 MS patients and 95 healthy controls (HCs) from the Amsterdam MS cohort, of which a subset was also seen after approximately 5-years of follow-up (229 patients and 58 HCs) (Eijlers et al., 2018). Detailed demographic data is presented in Table 1. All participants underwent an extensive MR imaging protocol at baseline (between 2008 and 2012), physical and cognitive evaluations at baseline and follow-up (2014 and 2017). Educational level was determined using a scale from 1 (unfinished primary school) to 7 (university degree or higher) (Verhage, 1964).

**Table 1:** Patient characteristics. Table 1 shows the patient characteristics at baseline and follow-up including demographic, clinical and imaging variables. Cognitively impaired (CI, two or more domains Z-scores below −2), mild cognitively impaired (MCI, at least two domains scored below Z = −1.5 and does not fulfill the CI criterion), or cognitively preserved (CP, all remaining patients).(Eijlers et al., 2017). ^a^mean, SD, independent samples *T*-test, ^b^ number, percentage, Chi-square test, ^c^ median, IQR, Mann-Whitney *U* test, ^d^ multivariate general linear model, covariates: sex, age and education. Abbreviations: BL: baseline; EDSS: expanded disability status scale; FU: follow-up; ml: milliliter; MS: multiple sclerosis; n: number; n.: nucleus; PPMS: primary progressive multiple sclerosis; RRMS: relapsing-remitting multiple sclerosis; SPMS: secondary progressive multiple sclerosis; TWT: 25-feet walk test; 9-HPT: 9-hole peg test.

		Patients (n = 331)	Healthy controls (n = 95)	*p*-value
**Baseline**				
	Age at BL (years)	48.1 (11.1)	45.8 (10.5)	0.073^a^
	Sex (% female)	225 (68.0)	55 (57.9)	0.068^b^
	Educational level BL	5 (4 – 6)	6 (4 – 7)	**0.012^c^**
	MS phenotype			
	−RRMS	242 (73.2)		
	−SPMS	53 (16.0)		
	−PPMS	36 (10.9)		
	Symptom duration	12.8 (7.0 – 20.9)		
	EDSS BL	3.0 (2.5 – 4.5)		
	Average cognition (z-score) BL	−0.80 (0.94)	0.00 (0.49)	**<0.001^a^**
	−CP	179 (54.1 %)		
	−MCI	65 (19.6 %)		
	−CI	87 (26.3 %)		
**Cerebellar volumes (in ml)**				
	Anterior lobe	18.90 (1.9)	19.54 (1.6)	**0.010^d^**
	Posterior lobe	128.91 (11.7)	133.47 (11.2)	**0.002^d^**
	I to IV	8.20 (0.87)	8.49 (0.76)	**0.009^d^**
	V	10.70 (1.08)	11.05 (0.87)	**0.016^d^**
	VI	22.84 (2.28)	23.60 (1.99)	**0.008^d^**
	Vermis VI	2.15 (0.23)	2.21 (0.24)	0.052^d^
	Crus I	32.76 (3.37)	33.75 (3.44)	**0.019^d^**
	Vermis Crus I	0.010 (0.005)	0.011 (0.006)	0.21^d^
	Crus II	23.16 (2.32)	24.07 (2.22)	**0.003^d^**
	Vermis Crus II	0.49 (0.06)	0.49 (0.06)	0.87^d^
	VIIb	12.33 (1.27)	12.79 (1.17)	**0.006^d^**
	Vermis VIIb	0.24 (0.03)	0.25 (0.03)	0.14^d^
	VIIIa	12.23 (1.20)	12.61 (1.14)	**0.020^d^**
	Vermis VIIIa	1.50 (0.18)	1.56 (0.17)	**0.015^d^**
	VIIIb	9.83 (1.02)	10.21 (1.02)	**0.006^d^**
	Vermis VIIIb	0.74 (0.09)	0.77 (0.09)	**0.029^d^**
	IX	7.80 (1.09)	8.25 (1.05)	**0.001^d^**
	Vermis IX	0.96 (0.12)	1.00 (0.12)	**0.014^d^**
	X	1.52 (0.21)	1.56 (0.19)	0.15^d^
	Vermis X	0.34 (0.04)	0.34 (0.04)	0.74^d^
	n. dentate	4.08 (0.39)	4.32 (0.33)	**<0.001^d^**
	n. interposed	0.55 (0.06)	0.59 (0.05)	**<0.001^d^**
	n. fastigial	0.098 (0.014)	0.103 (0.013)	**0.009^d^**
	Lesions	0.011 (0.034)		
**Follow-up**		**n = 229**	**n = 58**	
	Time interval BL – FU	4.5 (4.3 – 5.3)	5.3 (4.5 – 6.5)	**<0.001^c^**
	Age at BL (in years)	47.6	45.9	0.30^a^
	Sex (% female)	155 (67.7)	30 (51.7)	**0.023^b^**
	Educational level at BL	5 (4 – 6)	6 (4 – 7)	**0.016^c^**
	MS phenotype FU			
	− RRMS	156 (47.1 %)		
	− SPMS	54 (16.3 %)		
	− PPMS	19 (5.7 %)		
	EDSS FU	3.5 (2.5 – 4.5)		
	EDSS change / year	0.09 (0.0 – 0.2)		
	Average cognition	−1.01 (0.92)	0.01 (0.54)	**<0.001^a^**
	Annual cognitive decline (RCI/year)	−0.044 (0.103)	0.002 (0.064)	**0.001^a^**

This study was approved by the institutional ethics review board of the Amsterdam UMC (location VUmc) and all participants gave written informed consent prior to participation.

### 2.2 Clinical evaluation

Physical disability was measured using the Expanded Disability Status Scale (Kurtzke, 1983) (EDSS). Disability progression was measured using change in EDSS scores between baseline and follow-up divided by the time between visits. Additionally, EDSS progression as used in previous clinical trials was calculated for descriptive purposes and defined as an increase in EDSS of 1.5, 1.0 or 0.5 in case of a baseline EDSS of 0, 1–5.5 or ≥ 6.0, respectively) (Uitdehaag, 2014).

An expanded Brief Repeatable Battery of Neuropsychological tests (BRB-N) (Kurtzke, 1983) was used to measure cognitive functioning (Rao, 1990) including 7 domains: executive functioning (concept shifting test), information processing speed (symbol digit modalities test), attention (Stroop color-word test), working memory (memory comparison test), verbal fluency (word list generation test) visuospatial memory (spatial recall test) and verbal memory (selective reminding test). Raw test scores were corrected for effects of age, sex and education by converting them into Z-scores per domain based on baseline HCs cognitive scores. Baseline average cognitive functioning was calculated using the average of all corrected Z-scores.

The change in cognitive functioning between baseline and follow-up was calculated as a yearly cognitive decline rate, as described previously (Iverson, 2001), using the modified practice-adjusted reliable change index (RCI). This RCI corrects for learning effects in HCs and was calculated for each cognitive domain, and divided by the interval duration between baseline and follow-up to create an annualized cognitive domain decline rate (Eijlers et al., 2018). These rates were averaged across domains to compute an average annualized cognitive decline rate.

### 2.3 Imaging acquisition and processing

All patients underwent brain MRI at 3 T (General Electric, Milwaukee, WI, USA) using an eight-channel phased-array head coil. For volumetric measurements, a 3D T1-weighted fast spoiled gradient-echo sequence (repetition time (TR) = 8 ms, echo time (TE) = 3 ms, inversion time(IT) = 450 ms, flip angle 12 degrees, 1.0 mm sagittal slices, 0.9 × 0.9 mm in-plane resolution) and a 3D T2-weighted fluid attenuated inversion recovery (FLAIR) sequence (TR = 8000 ms, TE = 125 ms, IT = 2350 ms, 1.2 mm sagittal slices, 1.0x1.0 mm in-plane resolution). White matter (WM) lesions were automatically segmented on FLAIR and lesion maps were then transferred to 3DT1 images for lesion filling with LEsion Automated Pre-processing (LEAP), as previously described (Eijlers et al., 2018).

Cerebellar lobule segmentation was performed on the lesion-filled 3D T1-weighted images using the Spatially Unbiased Infratentorial Toolbox (SUIT) (Diedrichsen et al., 2009) in MATLAB R2012a using SPM12. The cerebellar segmentations can be viewed online using the atlas viewer provided by Diedrichsen lab (https://www.diedrichsenlab.org/imaging/AtlasViewer/). The order of cerebellum parcellation and segmentation was: 1. Whole-brain normalization, 2. Cerebellum isolation resulting in grey matter (GM) and WM probability maps, 3. Mapping and back-projecting with the SUIT-specific cerebellum atlas template. All segmentations were then multiplied with inverted cerebrospinal fluid masks to remove any residual segmentation errors, and corrected for skull size by v-scaling, both derived from SIENAX (FSL 5, https://fsl.fmrib.ox.ac.uk/fsl/).

**Fig. 1:**
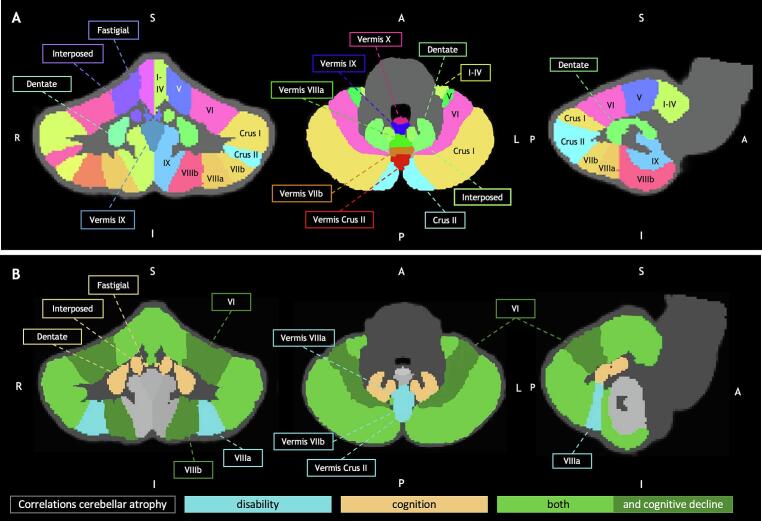
**Cerebellar segmentation and correlation to baseline and longitudinal clinical outcome.**Fig. 1A illustrates the individual lobules and nuclei of the cerebellum. The anterior lobe includes lobules I to V and the posterior lobe includes lobules VI to X. Fig. 1B illustrates the correlation of regional atrophy with disability (light blue), cognition (copper) or both (green). Dark green illustrates the correlation of regional atrophy with both disability and cognition, as well as with cognitive decline. (For interpretation of the references to color in this figure legend, the reader is referred to the web version of this article.)

The cerebellum was parcellated in an anterior lobe (lobules I-V), posterior lobe (lobules VI-X) and three nuclei (dentate, interposed, fastigial). The anterior and posterior lobes were further sub-segmented into 10 individual lobules (I-X) and 5 vermis lobules (vermis I-X); left and right segmentations were added. In addition, as is standard for SUIT, lobule VII was separated in lobule VIIb, crus I and crus II, lobule VIII in lobules VIIIa and VIIIb (Fig. 1a).

## 3 Statistical analysis

### 3.1 Group differences in demographics and clinical variables

Statistical analysis was performed using SPSS 22.0 (IBM, Chicago, USA). Normality of data was checked using visual inspection of histograms in combination with Kolmogorov-Smirnov testing. Differences between MS patients and HC for baseline demographic and clinical data were evaluated using parametric (independent samples *t*-test) and non-parametric tests (Mann-Whitney *U* test and chi-square test) as appropriate.

### 3.2 Cross-sectional and longitudinal correlations

Firstly, we aim to screen for regions of possible interested to evaluate further in the main correlation analyses. As such, cerebellar volumes were compared between HC and patients using multivariate general linear models with sex, age and education as covariates.

Secondly, these regions with a difference in MS were further assessed using partial correlations looking at relations with disability (baseline EDSS score) and cognitive functioning (baseline average cognition Z-score), with sex, age and education as covariates; a Bonferroni correction for multiple testing was applied (*p*-value*21, i.e. the total number of cerebellar lobules and nuclei). Level of significance was set at *p*-value < 0.05. Cerebellar regions correlating with average cognition were further explored for their relations with the cognitive domains at baseline (*p*-value*21, i.e. the total number of cerebellar lobules and nuclei).

For follow-up measurements of disability progression (EDSS change per year) and cognitive decline (RCI per year), those cerebellar volumes showing significant cross-sectional correlations were also correlated to clinical progression measures using partial correlations, again correcting for age, sex and education.

## 4 Results

### 4.1 Clinical characteristics at baseline and follow-up

At baseline 242/331 patients (73.2 %) were classified as relapsing-remitting MS, 53/331 (16.0 %) with secondary progressive MS and 36/331 (10.9 %) with primary progressive MS. Median symptom duration was 12.8 years (IQR 7.0–20.9) and baseline EDSS was 3.0 (IQR 2.5–4.5). Disease-modifying treatments (DMT) included glatiramer acetate (n = 27), natalizumab (n = 26), β-interferons (n = 101), fingolimod (n = 6), other immunosuppressive therapy (n = 12), unknown (n = 30), and of 36 patients medication use was not recorded and 93 patients were not on any DMT. Average cognitive functioning was significantly lower for patients compared to HCs (Z = −0.8, p < 0.001). Among MS patients, 179 (54.1 %) patients were cognitively preserved, 65 (19.6 %) had MCI and 87 (26.3 %) were cognitively impaired. Patients and controls were age- and sex-matched, but the level of education was significantly lower in patients (median 5, IQR 4–6) compared to HCs (median 6, IQR 4–7). See Table 1 for all demographic, clinical and imaging characteristics.

A total of 229 patients (mean interval 4.8 years, SD 0.8) and 58 HCs (mean interval 5.5 years, SD 1.1) returned for a follow-up visit. Compared to HCs, patients in this subgroup also had a significant lower baseline educational level and female/male ratio was different (see Table 1). As described in Table 1, in the MS group, 79 patients (34.5 %) showed EDSS progression from 3.0 to 3.5 (median EDSS change 0.09) and 26.6 % of patients showed cognitive decline on at least 2 domains.

### 4.2 Cerebellar atrophy and clinical correlations

Baseline cerebellar lesion volume did not correlate significantly with EDSS (r = 0.060, *p* = 0.279) or cognition (r = −0.067, *p* = 0.225).

Compared to HCs, cerebellar lobules and nucleus volumes were significantly lower in the anterior (lobules I to V, *p* = 0.003) and posterior lobes (lobules VI tot X, *p* = 0.001) as well as in the dentate (*p* < 0.001), interposed (p < 0.001) and fastigial (*p* = 0.005) nuclei in patients. Most sub-segmented lobule volumes were also significantly lower in MS patients than in HCs (Table 1).

Baseline partial correlations (Table 2) showed a significant correlation for the anterior and posterior lobe with both disability (anterior lobe r = −0.30, *p* < 0.001; posterior lobe r = −0.30, *p* < 0.001) and cognitive performance (anterior lobe r = 0.20, *p* = 0.007; posterior lobe r = 0.22, *p* = 0.001).

**Table 2:** Significant correlations between cerebellar volumes and clinical outcome measures. Table 2 shows the correlations of the cerebellar volumes (clustered and regional) with disability (EDSS), cognitive performance (average cognition) and the cognitive domains (sex, age and education as covariates; Bonferroni correction for multiple testing). Lines that were left blank were not significant. Δ year: yearly change. x: significant correlation between cognitive domain and cerebellar volume. Abbreviations: Att: attention; BL: baseline; EDSS: Expanded disability status scale; EF: executive functioning; IPS: information processing speed; R: correlation coefficient; RCI: reliable change index; vf: verbal fluency; vsm: visuospatial memory; WoM: working memory.

		Disability				Cognitive performance				Cognitive domains						
Cerebellar volume		EDSS BL		EDSS (Δ year)		Average cognition BL		RCI (Δ year)		IPS	WoM	VM	Att	EF	VF	VSM
		R	*p*-value	R	*p*-value	R	*p*-value	R	*p*-value							
**Clustered volumes**																
	Anterior lobe	−0.303	<0.001			0.197	0.007			x	x					
	Posterior lobe	−0.300	<0.001			0.219	0.001	0.136	0.041	x	x	x				
	Dentate nucleus					0.241	<0.001			x		x	x			
	Interposed nucleus					0.247	<0.001			x		x		x	x	x
	Fastigial nucleus					0.176	0.028									
**Regional volumes**																
	I to IV	−0.265	<0.001			0.192	<0.001			x	x					
	V	−0.317	<0.001			0.191	0.011									
	VI	−0.326	<0.001			0.217	0.002	0.153	0.021	x						
	Vermis VI	−0.274	<0.001			0.221	0.001	0.146	0.028	x	x					
	Crus I	−0.306	<0.001			0.184	0.017									
	Vermis Crus I															
	Crus II	−0.196	0.008			0.208	0.003			x	x					
	Vermis Crus II	−0.182	0.019													
	VIIb	−0.251	<0.001			0.214	0.002			x	x	x				
	Vermis VIIb	−0.267	<0.001													
	VIIIa	−0.249	<0.001													
	Vermis VIIIa	−0.214	0.002													
	VIIIb	−0.237	<0.001			0.178	0.025	0.138	0.039	x	x					
	Vermis VIIIb	−0.208	0.003													
	IX															
	Vermis IX															
	X	−0.221	0.001													
	Vermis X															

In progressive MS, both cognition and disability significantly correlated with the anterior lobe (cognition, r = 0.357, *p* < 0.001; disability, r = −0.372, *p* < 0.001) and posterior lobe (cognition, r = 0.362, *p* < 0.001; disability, r = −0.394, *p* < 0.001). In RRMS, EDSS correlated to significantly to the anterior (r = −0.224, *p* < 0.001) and posterior lobe (r = −0.230, *p* < 0.001), whereas cognition correlated significantly to the posterior (r = 0.145, *p* = 0.025), but not anterior lobe (r = 0.122, *p* = 0.060). In HC, cognition did not correlate significantly with the anterior lobe (r = 0.050, *p* = 0.633) or posterior lobe (r = 0.022, *p* = 0.834).

Volumes of individual nuclei were only correlated to cognitive performance (dentate nucleus r = 0.24, *p* < 0.001; interposed nucleus r = 0.25, *p* < 0.001; fastigial nucleus r = 0.18, *p* = 0.028).

Sub-segmented individual lobules showing atrophy were correlated to clinical scores at baseline (see Fig. 1b and Table 2 for more details). Volumes of lobules VIIIa and X as well as of vermis Crus II, vermis VIIb, vermis VIIIa and vermis VIIIb were only related to baseline EDSS. Volumes of lobules I to IV, V, VI, Crus I, Crus II, VIIb, VIIIb as well as vermis VI, were correlated to both baseline disability and cognition. None of the regional volumes, apart from the abovementioned nuclei, were uniquely related to cognition.

Correlations per cognitive domain (Table 2) showed that executive functioning, verbal fluency and visuospatial memory were related to atrophy of the interposed nucleus only, while verbal memory was related to crus II, VIIb, dentate and the interposed nuclei. Information processing speed and working memory were related to atrophy of lobules I to IV, crus II, VIIb, VIIIb and vermis VI, and finally information processing speed was related to atrophy of lobule VI, dentate and interposed nuclei.

Longitudinally, of all variables showing clinical relevance at baseline, only the posterior lobe was correlated to cognitive change over time (Table 2). Within this lobe, atrophy of three individual cerebellar lobules correlated with cognitive decline: VI and VIIIb and vermis VI. None of the cerebellar regions correlated to EDSS change per year (Table 2).

## 5 Discussion

The impact of regional cerebellar atrophy on clinical progression, particularly in relation to cognitive function, remains unclear. Therefore, in the present study we determined regional cerebellar atrophy patterns and related these to cross-sectional and longitudinal measures of disability and cognition in MS (D’Ambrosio et al., 2016, Henry et al., 2008, Iglói et al., 2015). Cross-sectionally, disability and cognition correlated strongly to multiple cerebellar regions in both anterior and posterior lobes. Some cerebellar cortical regions were specifically correlated to EDSS, whereas cognitive dysfunction was uniquely correlated to the deep nuclei (interposed, dentate and fastigial nucleus). Longitudinal results indicate that posterior lobe atrophy, and more specifically lobule VI, VIIIb and vermis VI atrophy, correlated to cognitive decline. No baseline atrophy correlations were found for disability progression.

### 5.1 Regional cerebellar atrophy and disability

Our results showed that regions in both the anterior and posterior lobe were related to EDSS, while previous studies mostly implicate anterior regions. The involvement of posterior areas could be explained by our finding of atrophy in lobule VI and VIII that are known to be connected to cerebral motor areas (Buckner et al., 2011, King et al., 2019, Stoodley and Schmahmann, 2010). As such, it seems logical that atrophy of these individual cerebellar areas could lead to decreased motor function. However, previous studies determining the relation between (regional) cerebellar atrophy in MS and disability were inconclusive (Cocozza et al., 2017, D’Ambrosio et al., 2016, Henry et al., 2008). For instance, one study showed that only the anterior lobe and brain T2 lesion volumes predicted EDSS scores and 9-hole peg test (9-HPT) performance (D’Ambrosio et al., 2016). In fact, one other study which also used SUIT for cerebellar segmentation did not find any relation between individual lobules and EDSS, and only relations for cerebellar lesion volumes, but not atrophy, and 9-HPT (Cocozza et al., 2017). These contradictory findings could be partly due to the size of region investigated, segmenting larger versus smaller lobules, and/or cohorts studied ranging from RRMS (D’Ambrosio et al., 2016) to progressive MS (Cocozza et al., 2017) or a combination as done in this study. Previous studies have shown different mechanisms underlying atrophy patterns in the cerebrum between relapsing and progressive MS, indicating that possibly progressive MS features more primary neurodegenerative characteristics, which remains unclear for the cerebellum. In this study we did observe subtype differences, with correlations between lobule atrophy and cognition and disability being slightly stronger in progressive patients compared to RRMS. However, cerebellar atrophy patterns in MS subtypes should be researched in more depth in future studies, preferably including larger samples sizes**.** In addition, disability is also importantly driven by spinal cord atrophy, this might be the reason why some studies do not show a specific result for the cerebellum. In addition, cerebellar research could be advanced further using more sophisticated clinical measures of (cerebellar) motor function such as laboratory gait assessments.

Regionally, several regions correlated with EDSS, with lobule V, VI and crus I demonstrating strongest correlations. Lobule V is not surprising, as part of the anterior lobe, has been related to normal motor function in many studies (Buckner et al., 2011, King et al., 2017, Stoodley et al., 2012, Stoodley and Schmahmann, 2010). In addition, histological tracing studies showed direct afferent and efferent connections between lobule V and VI with the primary motor cortex in the cerebrum (Kelly & Strick, 2003). Slightly more unexpected, we found a relation between crus I and EDSS, while most studies showed that crus I is mostly related to cognition (Buckner et al., 2011, Stoodley and Schmahmann, 2010). However, a previous study, did report reduced functional connectivity of crus I (and dentate nucleus) related to higher EDSS and ataxia scores (Dogonowski et al., 2014). This may be explained by the cognitive aspects of crus I which could have an indirect effect on mobility (for instance due to its role in spatial navigation) (Iglói et al., 2015). Similarly, besides motor involvement, lobule VI was indicated to play a role in cognitive functions such as memory, language and executive function (Buckner et al., 2011). The precise mechanisms by which these cerebellar structures specifically relate to disability, higher level cognition, and their interplay in MS remains unclear.

Longitudinally, no correlations were found for the yearly change in EDSS score. A possible reason is that this outcome measure is too coarse, which is a common topic of continued debate in the field. Multiple, more sophisticated outcome measures may be necessary to detect different types and more subtle signs of cerebellar dysfunction such as disturbances in coordination, balance, upper and lower limb dysfunction. Although previous studies used more specific disability measures such as lower limb function (25-foot walk test) or upper limb function (9-hole peg test), results on the relation with (regional) cerebellar atrophy have thus far been inconclusive (Anderson et al., 2009, Cocozza et al., 2017, D’Ambrosio et al., 2016, Henry et al., 2008).

### 5.2 Regional cerebellar atrophy and cognition

Baseline cognitive performance was related to both anterior and posterior lobe atrophy, with posterior lobe demonstrated strongest relation and involvement in cognitive decline over time. The posterior lobe has been indicated to play a role in cognition in previous literature (Cocozza et al., 2017, D’Ambrosio et al., 2016, Moroso et al., 2017, D’Ambrosio et al., 2016, Eijlers et al., 2018, Iglói et al., 2015). Regionally, atrophy of vermis VI and Lobule VI were most strongly related to baseline cognition as well as cognitive decline. The value of lobule VI has been demonstrated in a cross-sectional study (Cocozza et al., 2017) but not in a longitudinally study design. Lobule VI and vermis VI have also been related to normal cognitive performance (Cocozza et al., 2017), and specifically vermis VI was previously related to normal information processing speed, verbal and working memory and executive functioning (King et al., 2019, Nettekoven et al., 2024, Stoodley and Schmahmann, 2009). A study in MS on the cerebellum and information processing speed, highlighted the relevance of vermis VI atrophy in MS (Moroso et al., 2017), which is in line with our results showing a relation between vermis VI and information processing speed.

The deep nuclei of the cerebellum were specifically related to baseline cognition, not EDSS. The dentate, interposed and fastigial nucleus have rarely been implicated in MS research previously. One study did report atrophy of the red nucleus, which receives input from the cerebellum, related to volume loss of the interposed nucleus in MS, but did not investigate relations to clinical disability (Margoni et al., 2020). The interposed nucleus has been related to normal motor (Dimitrova et al., 2006) as well as cognitive functioning (Lu et al., 2012) with different techniques. Interestingly, in this study the interposed nucleus was related to most cognitive domains with strongest relations with verbal memory, working memory and information processing speed. Existing studies often focused on information processing speed (D’Ambrosio et al., 2016, Moroso et al., 2017) but did not include a broad range of cognitive domains. In addition, our results on the dentate nucleus are in line with other studies finding activation of the dentate nucleus during different cognitive tasks (Kim et al., 1994, Thürling et al., 2011). Such information on the involvement of cerebellar nuclei in cognition is new, as previous work in this area remains scarce (D’Ambrosio et al., 2016, Iglói et al., 2015).

### 5.3 Limitations and suggestions for future research

Although this is a well-documented, longitudinal cohort, some limitations must be addressed. The thin and highly folded cortical layer of the cerebellum affects the segmentation of the individual lobules and nuclei, future studies may need even better methods of segmenting the cerebellum. In addition, compared to the clinical field strength (3 T) used in this study, with ultra-high field MRI (7 T) the cerebellum can be imaged at higher spatial resolutions (Priovoulos et al., 2023), potentially allowing for better characterization of cerebellar damage and improvement of segmentation accuracy of the small substructures assessed in this study. We acknowledge identifying specific mechanisms related to clinical decline remains difficult, as those with more severe neurodegeneration in general are most likely most prone to progress. Therefore, further research incorporating longitudinal atrophy measurements alongside clinical decline is needed to study disease progression in more detail*.* Additionally, implementing functional and structural connectivity with key supratentorial motor networks could help clarifying the role of the cerebellum in disability progression and cognitive decline.

## 6 Conclusion

In this study we found that regional cerebellar atrophy was related to both physical disability and cognitive impairment, especially verbal and working memory as well as information processing speed. Atrophy in specific regions was related to physical disability only or both disability and cognition, whereas atrophy of the cerebellar nuclei related to cognitive performance only. Longitudinally, atrophy of the posterior lobe (lobule VI, VIIIb and vermis VI) correlated with cognitive decline, while no correlations were found for physical disability progression. Future studies including longitudinal imaging data are now needed to further elucidate the impact of the development of regional cerebellar atrophy on clinical progression in MS.

## Ethical considerations

This study was approved by the institutional ethics review board of the Amsterdam UMC, the Netherlands (location VUmc).

## Consent to participate

All participants gave written informed consent prior to participation.

## Funding statement

This study was supported by the Dutch MS research foundation, grants 08-650, 13-820, 09-358d and 14-358e. FB is supported by the NIHR biomedical research center at 10.13039/501100008721UCLH.

## CRediT authorship contribution statement

**Myrte Strik:** Writing – review & editing, Writing – original draft, Visualization, Project administration, Methodology, Investigation, Formal analysis, Data curation, Conceptualization. **Iris Dekker:** Writing – review & editing, Writing – original draft, Visualization, Resources, Project administration, Methodology, Investigation, Funding acquisition, Formal analysis, Data curation, Conceptualization. **Aurélie Ruet:** Writing – review & editing, Conceptualization. **Hanneke E. Hulst:** Writing – review & editing, Conceptualization. **Mike P. Wattjes:** Writing – review & editing, Resources, Funding acquisition, Conceptualization. **Frederik Barkhof:** Writing – review & editing, Resources, Funding acquisition, Conceptualization. **Bernard M.J. Uitdehaag:** Writing – review & editing, Resources, Funding acquisition, Conceptualization. **Joep Killestein:** Writing – review & editing, Resources, Funding acquisition, Conceptualization. **Menno M. Schoonheim:** Writing – review & editing, Writing – original draft, Supervision, Resources, Project administration, Methodology, Investigation, Funding acquisition, Formal analysis, Data curation, Conceptualization.

## Declaration of competing interest

The authors declare the following financial interests/personal relationships which may be considered as potential competing interests: M. Strik receives research support from Dutch MS Research Foundation. I. Dekker has nothing to declare. A. Ruet reports consultancy fees, speaker fees, travel grants, research grants (non-personal), or honoraria approved by the institutions from Novartis, Biogen, Genzyme, Roche, Alexion, BMS, Merck. H.E. Hulst serves on the editorial board of Multiple Sclerosis Journal, receives research support from the Dutch MS Research Foundation and the Dutch Research Council. She has served as a consultant for or received research support from Atara Biotherapeutics, Biogen, Novartis, Celgene/Bristol Meyers Squibb, Sanofi Genzyme, MedDay and Merck BV. M.P. Wattjes reports personal fees from Biogen (Consultancy, speaker fee), personal fees from Novartis Consultancy, speaker fee), personal fees from Roche (Consultancy, speaker fee), personal fees from Celgene (Consultancy, speaker fee), personal fees from IXICO (Consultancy, speaker fee), personal fees from Sanofi Genzyme (speaker fee), personal fees from Bayer Healthcare (speaker fee), personal fees from Biologix (speaker fee), personal fees from Genilac (speaker fee), personal fees from Merck Serono (Consultancy, speaker fee), personal fees from Icometrix (consultancy), personal fees from Alexion (consultancy and speaker fee), personal fees from Eisai (consultancy and speaker fee), personal fees from Lilly (consultancy and speaker fee), personal fees from New Bridge Pharma (speaker fee) outside the submitted work. F. Barkhof is a member of the steering committee or Data Safety Monitoring Board for Biogen, Merck, Eisai and Prothena. Advisory board member for Combinostics, Scottish Brain Sciences, Alzheimer Europe. Consultant for Roche, Celltrion, Rewind Therapeutics, Merck, Bracco. Research agreements with ADDI, Merck, Biogen, GE Healthcare, Roche. Co-founder and shareholder of Queen Square Analytics LTD. B.M.J. Uitdehaag reports research consultancy fees from Immunic Therapeutics. J. Killestein received research grants for multicentre investigator initiated trials DOT-MS trial, ClinicalTrials.gov Identifier: NCT04260711 (ZonMW) and BLOOMS trial (ZonMW and Treatmeds), ClinicalTrials.gov Identifier: NCT05296161); received consulting fees for F. Hoffmann-La Roche Ltd, Biogen, Teva, Merck, Novartis and Sanofi/Genzyme (all payments to institution); reports speaker relationships with F. Hoffmann-La Roche, Biogen, Immunic, Teva, Merck, Novartis and Sanofi/Genzyme (all payments to institution); adjudication committee of MS clinical trial of Immunic (payments to institution only). M.M. Schoonheim serves on the editorial board of Neurology, Multiple Sclerosis Journal and Frontiers in Neurology, receives research support from the Dutch MS Research Foundation, Eurostars-EUREKA, ARSEP, Amsterdam Neuroscience and ZonMW (Vidi grant, project number 09150172010056) and has served as a consultant for or received research support from Atara Biotherapeutics, Biogen, Celgene/Bristol Meyers Squibb, EIP, Sanofi, MedDay and Merck.

## Data Availability

Anonymized data and code, not published in the article, will be shared on reasonable request from a qualified investigator.
