# Supplementary material for: Regional cerebellar atrophy related to disability and cognitive progression in multiple sclerosis
Source: Neuroimage Clin. 2025 Apr 23;46:103792. doi: 10.1016/j.nicl.2025.103792 (PMC12264214; doi:10.1016/j.nicl.2025.103792)
Supplement: Supplementary Data 1 [file mmc1.docx]

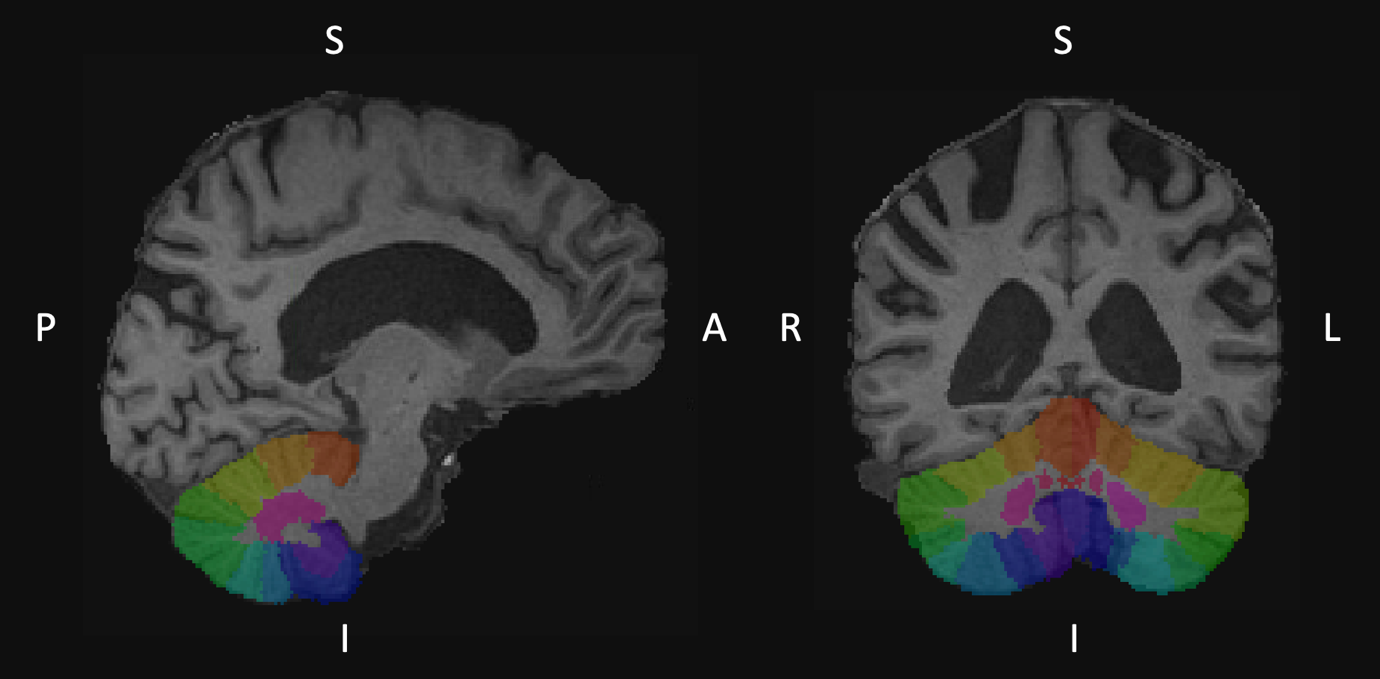
**Supplementary Figure 1. Cerebellar segmentation.** Example of cerebellar segmentation in an MS patient. The SUIT segmentation is overlayed on the T1.
